# A noncommutative catenoid

**DOI:** 10.1007/s11005-017-1042-z

**Published:** 2018-01-04

**Authors:** Joakim Arnlind, Christoffer Holm

**Affiliations:** Department of Mathematics, Linköping University, 581 83 Linköping, Sweden

**Keywords:** Noncommutative catenoid, Noncommutative Riemannian geometry, Noncommutative minimal surface, Noncommutative curvature, 46L87, 58B34

## Abstract

A noncommutative algebra corresponding to the classical catenoid is introduced together with a differential calculus of derivations. We prove that there exists a unique metric and torsion-free connection that is compatible with the complex structure, and the curvature is explicitly calculated. A noncommutative analogue of the fact that the catenoid is a minimal surface is studied by constructing a Laplace operator from the connection and showing that the embedding coordinates are harmonic. Furthermore, an integral is defined and the total curvature is computed. Finally, classes of left and right modules are introduced together with constant curvature connections, and bimodule compatibility conditions are discussed in detail.

## Introduction

In recent years, there has been great progress in understanding Riemannian aspects of noncommutative geometry and its relation to topology. For instance, the scalar curvature defined via the heat kernel has been computed for noncommutative tori and a noncommutative version of the Gauss–Bonnet theorem has been established (see e.g [9, 11, 15, 16]). In parallel, one has investigated the role of the Levi–Civita connection and the curvature tensor, in order to understand to what extent classical geometrical concepts remain relevant in noncommutative geometry (see e.g [1, 4–6, 8, 13, 14, 19]). In contrast to the approach via the heat kernel, much of this work has not been carried out in the setting of $C^*$-algebras and spectral triples, but rather taking a less analytical, and more algebraical, point of view, constructing curvature through a “bottom-up” approach starting from a hermitian form on a (projective) module. In the future, it will be interesting to see how these different approaches may be reconciled. Even though a lot of progress has been made it is not completely clear what kind of assumptions that are needed in order to find a unique Levi–Civita connection and what kind of properties (e.g. symmetries) that one should expect. Therefore, it is useful to consider particular examples to understand what one might (might not) expect in the general case.

Another motivation comes from the theory of minimal surfaces. Whereas the classical theory is by know well developed (although many interesting questions are still open), its noncommutative analogue is in an early stage. Several authors have approached noncommutative minimal submanifolds from different perspectives (see e.g [2, 12, 18]) but a general framework is still missing. The catenoid is one of the most well-known minimal surfaces in Euclidean space, and it is interesting to understand how its properties manifest themselves in noncommutative geometry. Note that related quantum catenoids have been considered, although not primarily from a geometrical point of view [2, 3].

In this note we shall construct a noncommutative algebra $\widehat{\mathcal {C}}_\hbar$ that is closely related to the classical catenoid, which is a (noncompact) minimal surface embedded in ${\mathbb {R}}^3$. The algebra $\widehat{\mathcal {C}}_\hbar$ is not a $C^*$-algebra in any natural way, and typical representations are given by unbounded operators. However, the algebraic structure is quite appealing and in many ways similar to the noncommutative torus, a fact we shall employ to find several natural constructions.

The paper is organized as follows: In Sect. 2 the algebra $\widehat{\mathcal {C}}_\hbar$, together with a set of derivations, is introduced and a few basic properties are established. Section 3 introduces a natural module of vector fields and proves that given a metric there exists a unique torsion-free connection which is compatible with the metric and the complex structure. Finally, Sect. 4 introduces an integral and computes the total curvature of the metric, and Sect. 5 studies bimodules together with constant curvature connections.

## The catenoid algebra

In this section we start from a parametrization of the classical catenoid and use the Weyl algebra in order to find a natural definition of a noncommutative catenoid. A parametrization of the catenoid embedded in ${\mathbb {R}}^3$ is given by

$$\begin{aligned} \vec {x}(u,v) = \big (x^1(u,v),x^2(u,v),x^3(u,v)\big ) = \big (\cosh (u)\cos (v),\cosh (u)\sin (v),u\big ) \end{aligned}$$

for $-\infty<u<\infty$ and $0\le v\le 2\pi$. The algebra generated by the functions $x^1,x^2,x^3$ can in principle also be generated by *u*, $e^{\pm u}$ and $e^{\pm iv}$. Now, starting from the Weyl algebra, consisting of two hermitian generators *U* and *V* satisfying

$$\begin{aligned} {[}U,V]=i\hbar \mathbb {1}, \end{aligned}$$

we shall construct an algebra generated by *U*, *R* and *W*, corresponding (formally) to *U*, $e^U$ and $e^{iV}$ respectively. Guided by the Baker–Campbell–Hausdorff formula, giving e.g.

$$\begin{aligned} RW = e^{U}e^{iV}=e^{U+iV+\frac{1}{2}[U,iV]}=e^{iV+U+\frac{1}{2}[iV,U]-[iV,U]} =e^{-\hbar }e^{iV}e^U=e^{-\hbar }WR, \end{aligned}$$

as well as the formal expansions of $e^U$ and $e^{iV}$ as power series, one introduces the following relations 
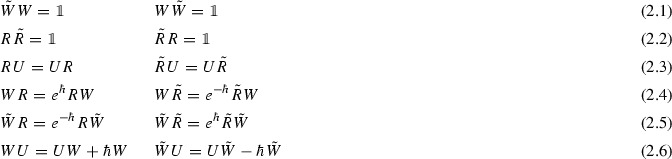
 where $\tilde{R}$ and $\tilde{W}$ have been introduced, representing the inverses of *R* and *W*.

### Definition 2.1

Let ${\mathbb {C}}\langle U,R,\tilde{R},W,\tilde{W}\rangle$ be the free associative unital algebra on the letters $U,R,\tilde{R},W,\tilde{W}$ and let $I_\hbar$ be the two-sided ideal generated by relations (2.1)–(2.6). We define $\mathcal {C}_{\hbar }$ as the quotient algebra

$$\begin{aligned} \mathcal {C}_{\hbar }={\mathbb {C}}\langle U,R,\tilde{R},W,\tilde{W}\rangle \slash I_\hbar . \end{aligned}$$

Next, we note that the relations (2.1)–(2.6) allows one to always order any element lexicographically (with respect to the alphabet $U,R,\tilde{R},W,\tilde{W}$, up to terms of lower total order) and, moreover, we prove that ordered monomials are linearly independent.

### Proposition 2.2

A basis for $\mathcal {C}_{\hbar }$ is given by

$$\begin{aligned} e^{\alpha j k} = U^\alpha R^j W^k \end{aligned}$$

for $\alpha \in {\mathbb {Z}}_{\ge 0}$ and $j,k\in {\mathbb {Z}}$, where $R^{-j}=\tilde{R}^j$ and $W^{-k}=\tilde{W}^k$.

### Proof

In the proof, we shall use the terminology of the Diamond Lemma [7] in order to show that $\{e^{\alpha jk}\}$ provides a basis for $\mathcal {C}_{\hbar }$. To this end we start by formulating relations (2.1)–(2.6) in the form of a reduction system: 
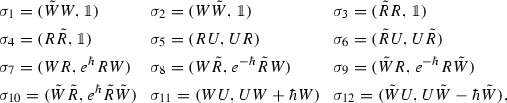
 for which we will use the notation $\sigma _i=(W_i,f_i)$. Let us set up a semi-group partial ordering on monomials in $U,R,\tilde{R},W,\tilde{W}$, which is compatible with the above reduction system. That is, a semi-group partial ordering such that if $\sigma _i=(W_i,f_i)$ then $W_i$ is strictly greater than every monomial in $f_i$. Let $p=X^1\cdots X^n$ and $q=Y^1\cdots Y^m$ be two monomials with $X^i,Y^i\in \{U,R,\tilde{R},W,\tilde{W}\}$. We say that $p<q$ if $n<m$ or if $n=m$ and *p* precedes *q* in lexicographic order with respect to the alphabet $U,R,\tilde{R},W,\tilde{W}$. It is easy to see that this does indeed define a semi-group partial ordering compatible with the above reduction system. Moreover, it is straightforward to check that the partial ordering satisfies the descending chain condition. Theorem 1.2 in [7] tells us that if all ambiguities in the above reduction system are resolvable, then a basis of $\mathcal {C}_{\hbar }$ is given by the irreducible monomials. That is, monomials which can not be reduced further by using the reduction system, replacing $W_i$ by $f_i$ for $i=1,\ldots ,12$. How do the irreducible monomials look like? It is clear that the monomial $U^\alpha R^j W^k$ is irreducible since it does not contain any of $W_1,\ldots ,W_{12}$. Moreover, there are no other irreducible polynomials since one may always use the reduction system to put monomials in lexicographic ordering, as well as replacing $R\tilde{R}$, $\tilde{R}R$, $W\tilde{W}$ and $\tilde{W}W$ by $\mathbb {1}$. Thus, it remains to prove that all ambiguities are resolvable. There are 20 ambiguities to be resolved:

$$\begin{aligned} (\tilde{W}W)\tilde{W}&=\tilde{W}(W\tilde{W}), (\tilde{W}W)R=\tilde{W}(WR), (\tilde{W}W)\tilde{R}=\tilde{W}(W\tilde{R}),\\ (\tilde{W}W)U&=\tilde{W}(WU), (W\tilde{W})R=W(\tilde{W}R), (W\tilde{W})\tilde{R}=W(\tilde{W}\tilde{R}),\\ (W\tilde{W})W&=W(\tilde{W}W), (W\tilde{W})U=W(\tilde{W}U), (\tilde{R}R)\tilde{R}=\tilde{R}(R\tilde{R}),\\ (\tilde{R}R)U&=\tilde{R}(RU), (R\tilde{R})U=R(\tilde{R}U), (R\tilde{R})R=R(\tilde{R}R),\\ (WR)\tilde{R}&=W(R\tilde{R}), (WR)U=W(RU), (W\tilde{R})R=W(\tilde{R}R),\\ (W\tilde{R})U&=W(\tilde{R}U), (\tilde{W}R)\tilde{R}=\tilde{W}(R\tilde{R}), (\tilde{W}RU)=\tilde{W}(RU),\\ (\tilde{W}\tilde{R})R&=\tilde{W}(\tilde{R}R), (\tilde{W}\tilde{R})U=\tilde{W}(\tilde{R}U), \end{aligned}$$

where the parenthesis mark which part of the monomial that is to be replaced by using the reduction system. It is straightforward to check that they are all resolvable, but let illustrate the procedure by explicitly checking $(\tilde{W}W)R=\tilde{W}(WR)$:

$$\begin{aligned} (\mathbb {1})R-\tilde{W}(e^{\hbar }RW)=R-e^{\hbar }\tilde{W}R W=R-e^{\hbar }e^{-\hbar }R\tilde{W}W=R-R=0. \end{aligned}$$

As previously stated, after showing that all ambiguities are resolvable, Theorem 1.2 in. [7] implies that the monomials $U^\alpha R^jW^k$ provide a basis for $\mathcal {C}_{\hbar }$. $\square$

We can make $\mathcal {C}_{\hbar }$ into a $*$-algebra by setting 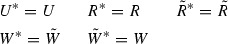 and noting that the set of relations (2.1)–(2.6) is invariant with respect to this involution. From now on we will use the more convenient notation $R^{-1}=\tilde{R}$ and $W^{-1}=\tilde{W}$. The next results gives a differential calculus on $\mathcal {C}_{\hbar }$, in direct analogy with the classical derivatives.

### Proposition 2.3

There exist hermitian derivations $\partial _{u},\partial _{v}\in {\text {Der}}(\mathcal {C}_{\hbar })$ such that 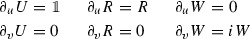 and $$[\partial _{u},\partial _{v}]=0$$.

### Proof

Since derivations are linear and satisfies the product rule, the relations in Proposition 2.3 completely determine the action of $\partial _u,\partial _v$ on $\mathcal {C}_{\hbar }$. However, in order to be well defined, one needs to check that the derivations respect the relations in $\mathcal {C}_{\hbar }$. Thus, one need to check that they are consistent with (2.1)–(2.6). For instance,

$$\begin{aligned} \partial _u(WR-e^{\hbar }RW)&=\partial _u(W)R+W\partial _u(R)-e^{\hbar }\partial _u(R)W-e^{\hbar }R\partial _u(W)\\&=WR-e^{\hbar }RW = 0. \end{aligned}$$

In the same way, one may check that $\partial _u$ and $\partial _v$ respect all the relations in the algebra. Moreover, one readily checks that the derivations are hermitian; for instance,

$$\begin{aligned} \partial _vW^*= \partial _vW^{-1}=-W^{-1}\partial _v(W)W^{-1}=-iW^{-1}=-iW^*=(iW)^*=(\partial _v W)^*, \end{aligned}$$

and analogous computations yield similar results for the remaining relations. $\square$

Let $\mathfrak {g}$ denote the (abelian) complex Lie algebra generated by $\partial _{u}$ and $\partial _{v}$, and introduce

$$\begin{aligned}&\partial = \tfrac{1}{2}(\partial _{u}-i\partial _{v})\\&\bar{\partial }= \tfrac{1}{2}(\partial _{u}+i\partial _{v}). \end{aligned}$$

For easy reference, let us write out 
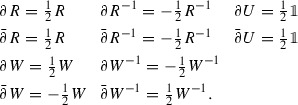
 In the classical setting, functions composed from $u,e^{\pm u},e^{\pm iv}$ make up a small subset of the smooth functions on the catenoid; for instance, even though $1+u^2$ is strictly positive for all $u\in {\mathbb {R}}$, there is no element corresponding to $1/(1+u^2)$ in the algebra. Thus, one would like to extend $\mathcal {C}_{\hbar }$ to include elements that correspond to more general functions. In this paper, our main concern is not to find the most general algebra for this purpose, but rather to take an opposite approach, where a minimal extension of $\mathcal {C}_{\hbar }$ is considered in order to develop the framework (although, it would be interesting to see how the results of [20] apply to the current situation). We will take an approach built on localization, using the Ore condition. Therefore, one starts by understanding the set of zero divisors.

### Proposition 2.4

The algebra $\mathcal {C}_{\hbar }$ has no zero-divisors.

### Proof

For every $a\in \mathcal {C}_{\hbar }$ one may write

$$\begin{aligned} a = \sum _{\alpha ,j,k}a_{\alpha jk}U^\alpha R^j W^k\qquad \end{aligned}$$

with $a_{\alpha j k}\in {\mathbb {C}}$, and we define the following integers

$$\begin{aligned} N(a)=&\max \{\alpha :\exists j,k\text { such that }a_{\alpha j k}\ne 0\}\\ J(a)=&\max \{j:\exists k\text { such that }a_{N(a) j k}\ne 0\}\\ K(a)=&\max \{k:a_{N(a)J(a)k}\ne 0\}. \end{aligned}$$

Now, assume that $ab=0$ with $a\ne 0$ and $b\ne 0$. From the relations (2.1)–(2.6) it follows that in the product *ab*, there is exactly one term proportional to

$$\begin{aligned} U^{N(a)+N(b)}R^{J(a)+J(b)}W^{K(a)+K(b)}, \end{aligned}$$

and the coefficient is given by $a_{N(a)J(a)K(a)}b_{N(b)J(b)K(b)}$. Now, since $\{U^\alpha R^jW^k\}$ is a basis for $\mathcal {C}_{\hbar }$ it follows that either $a_{N(a)J(a)K(a)}=0$ or $b_{N(b)J(b)K(b)}= 0$. However, this contradicts the assumption. Hence, if $ab=0$ then at least one of *a* and *b* has to be zero. $\square$

Next, we establish the Ore condition for $\mathcal {C}_{\hbar }$, which gives a condition for a non-trivial localization to exist.

### Lemma 2.5

For every $a,b\in \mathcal {C}_{\hbar }$, there exists $p,q\in \mathcal {C}_{\hbar }$ such that

$$\begin{aligned} ap = bq, \end{aligned}$$

and at least one of *p* and *q* is non-zero.

### Proof

The proof is a simple argument counting the number of equations and the number of variables in a set of linear equations (compare with the proof of a similar statement in the case of the Weyl algebra [17]). Let us assume that we are given $a,b\in \mathcal {C}_{\hbar }$, and define *N* to be the an integer such that

$$\begin{aligned} a_{\alpha jk}=b_{\alpha jk}=0 \end{aligned}$$

whenever at least one of $\alpha ,|j|,|k|$ is greater than *N*. Now, we have to find

$$\begin{aligned} p = \sum _{\alpha jk}p_{\alpha jk}U^\alpha R^j W^k\text { and } q = \sum _{\alpha jk}q_{\alpha jk}U^\alpha R^j W^k \end{aligned}$$

such that $ap-bq=0$. Let us choose *p* and *q* such that $p_{\alpha jk}= q_{\alpha jk}=0$ whenever $\alpha$, |*j*| or |*k*| is greater than *M*. This implies that *p* and *q* together has $2M(2M+1)^2$ coefficients to be determined. On the other hand, the equation $ap-bq=0$ gives rise to at most $(N+M)(2N+2M+1)^2$ linear equations in the coefficients $p_{\alpha jk}$ and $q_{\alpha jk}$, by looking at each basis element separately. Choosing $M=4N$ gives

$$\begin{aligned} \#\{\text {variables}\}-\#\{\text {equations}\} = 12N^3+56N^2+15N+1, \end{aligned}$$

which is $\ge 1$ for all $N\ge 0$. Since any homogeneous linear system, where the number of variables is strictly greater than the number of equations, has a non-zero solution, we conclude that there exists a solution where at least one of *p* and *q* is non-zero. $\square$

Together with Proposition 2.4, Lemma 2.5 implies that there exists a field of fractions $\mathcal {F}_\hbar$ for $\mathcal {C}_{\hbar }$ and, furthermore, that the inclusion map $\lambda :\mathcal {C}_{\hbar }\rightarrow \mathcal {F}_\hbar$ is injective (see e.g. [10]). The algebra $\mathcal {F}_\hbar$ is not particularly well suited as an analogue of the algebra of functions on the catenoid, since it contains many functions that are not well-defined at every point of the catenoid. Therefore, we will construct an extension of $\mathcal {C}_{\hbar }$ including inverses for a large class of polynomials.

Let $Z_{\hbar }(U,R)$ denote the commutative subalgebra of $\mathcal {C}_{\hbar }$ generated by $\mathbb {1}$, *U*, *R*, $R^{-1}$, and define a homomorphism (of commutative algebras) $\phi :Z_{\hbar }(U,R)\rightarrow C^{\infty }({\mathbb {R}})$ via

$$\begin{aligned} \phi (\mathbb {1})=1\qquad \phi (U) = u\qquad \phi (R)=e^{u}\qquad \phi (R^{-1})=e^{-u}. \end{aligned}$$

Define the following subset of $Z_{\hbar }(U,R)$:

$$\begin{aligned} Z_{\hbar }^{+}(U,R) = \{p\in Z_{\hbar }(U,R):|\phi (p)(u)|>0\text { for all }u\in {\mathbb {R}}\}. \end{aligned}$$

### Lemma 2.6

$Z_{\hbar }^{+}(U,R)$ is a multiplicative set.

### Proof

Let $p,q\in Z_{\hbar }^{+}$, which implies that $|\phi (p)|>0$ and $|\phi (q)|>0$. Since $\phi$ is a homomorphism, it follows that $|\phi (pq)| = |\phi (p)||\phi (q)|>0$. Hence $pq\in Z_{\hbar }^{+}(U,R)$. $\square$

In the following we would like to construct an algebra where all elements of $Z_{\hbar }^{+}(U,R)$ are invertible. To this end, let us recall a few basic results concerning noncommutative localization. Let $R,R'$ be rings, and let $\mathcal {S}$ be a subset of *R*. A homomorphism $f:R\rightarrow R'$ is called $\mathcal {S}$-inverting if *f*(*s*) is invertible for all $s\in \mathcal {S}$. A general construction gives the following result:

### Proposition 2.7

([10], Proposition 1.3.1) Given a ring *R* and a subset $\mathcal {S}\subseteq R$ there exists a ring $R_\mathcal {S}$ and an $\mathcal {S}$-inverting homomorphism $\iota :R\rightarrow R_S$ such that for every $\mathcal {S}$-inverting homomorphism $f:R\rightarrow R'$ there exists a unique homomorphism $g:R_S\rightarrow R'$ such that $f=g\circ \iota$.

However, the result does not provide any information on the kernel of $\iota$, which might be all of *R*. Thus, to guarantee a non-trivial localization one has to go one step further. First, let us consider the case when $R=\mathcal {C}_{\hbar }$ and $\mathcal {S}=\mathcal {C}_{\hbar }\backslash \{0\}$. Since $\mathcal {C}_{\hbar }$ satisfies the Ore condition and has no zero-divisors, one may conclude (cp. [10, Theorem 1.3.2]) that the inclusion map $\iota _1:\mathcal {C}_{\hbar }\rightarrow \mathcal {F}_\hbar =(\mathcal {C}_{\hbar })_\mathcal {S}$ is injective. Next, we let $\mathcal {S}=Z_{\hbar }^{+}(U,R)$ and consider $\widehat{\mathcal {C}}_\hbar =(\mathcal {C}_{\hbar })_{Z_{\hbar }^{+}(U,R)}$ together with $\iota :\mathcal {C}_{\hbar }\rightarrow \widehat{\mathcal {C}}_\hbar$. Now, consider the universal property of $\widehat{\mathcal {C}}_\hbar$ applied to $R'=\mathcal {F}_\hbar$ and $\iota _1:\mathcal {C}_{\hbar }\rightarrow \mathcal {F}_\hbar$. Clearly, $\iota _1$ is a $Z_{\hbar }^{+}(U,R)$-inverting map from $\mathcal {C}_{\hbar }$ to $\mathcal {F}_\hbar$. Hence, there exists a unique homomorphism $g:\widehat{\mathcal {C}}_\hbar \rightarrow \mathcal {F}_\hbar$ such that $\iota _1=g\circ \iota$. Since $\iota _1$ is injective it follows that $\iota$ is injective. In particular, this implies that the localization $\widehat{\mathcal {C}}_\hbar$ is non-trivial. Let us summarize the discussion in the following result.

### Proposition 2.8

There exists an algebra $\widehat{\mathcal {C}}_\hbar$ together with an injective $Z_{\hbar }^{+}(U,R)$-inverting homomorphism $\iota :\mathcal {C}_{\hbar }\rightarrow \widehat{\mathcal {C}}_\hbar$ such that for every ring $R'$ and every $Z_{\hbar }^{+}(U,R)$-inverting homomorphism $f:\mathcal {C}_{\hbar }\rightarrow R'$, there exists a unique homomorphism $g:\widehat{\mathcal {C}}_\hbar \rightarrow R'$ such that $f=g\circ \iota$.

For the algebra $\mathcal {C}_{\hbar }$, a basis was given by monomials of the form $U^\alpha R^jW^k$. For $\widehat{\mathcal {C}}_\hbar$ one may obtain a corresponding normal form, using the following result.

### Lemma 2.9

For every $p\in Z_{\hbar }^{+}(U,R)$ there exists $q\in Z_{\hbar }^{+}(U,R)$ such that

$$\begin{aligned} Wp=qW \end{aligned}$$

where $$q(U,R)=p(U+\hbar \mathbb {1},e^{\hbar }R)$$.

### Proof

Assume that $p\in Z_{\hbar }^{+}(U,R)$ and write

$$\begin{aligned} p = \sum p_{\alpha j}U^\alpha R^j \end{aligned}$$

which gives

$$\begin{aligned} Wp&= \sum p_{\alpha j}WU^\alpha R^j =\sum p_{\alpha j}(U+\hbar \mathbb {1})^\alpha W R^j\\&=\sum p_{\alpha j}(U+\hbar \mathbb {1})^\alpha (e^{\hbar }R)^j W =p(U+\hbar \mathbb {1},e^hR)W. \end{aligned}$$

Now, let us argue that $p(U+\hbar \mathbb {1},e^{\hbar }R)\in Z_{\hbar }^{+}(U,R)$. By construction, $\phi (p)(u)=p(u,e^u)$ which implies that

$$\begin{aligned} \phi \big (p(U+\hbar \mathbb {1},e^{\hbar }R)\big )(u)=p(u+\hbar ,e^{\hbar }e^u) =p(u+\hbar ,e^{u+\hbar })=\phi (p)(u+\hbar ). \end{aligned}$$

Since $|\phi (p)(u)|>0$ for all $u\in {\mathbb {R}}$, it follows that $|\phi (p)(u+\hbar )|>0$ for all $u\in {\mathbb {R}}$, which shows that $p(U+\hbar \mathbb {1},e^{\hbar }R)\in Z_{\hbar }^{+}(U,R)$. $\square$

From Lemma 2.9 one can derive

$$\begin{aligned}&Wp^{-1}=p(U+\hbar \mathbb {1},e^{\hbar }R)^{-1}W\\&W^{-1}p^{-1}=p(U-\hbar \mathbb {1},e^{-\hbar }R)^{-1}W^{-1} \end{aligned}$$

for $p\in Z_{\hbar }^{+}(U,R)$. Thus, using these relations, an element $a\in \widehat{\mathcal {C}}_\hbar$ can always be written as

$$\begin{aligned} a = \sum _{k\in {\mathbb {Z}}}a_kW^k \end{aligned}$$

with $a_k\in \mathcal {F}^+_\hbar (U,R)$, the commutative subalgebra of $\widehat{\mathcal {C}}_\hbar$ generated by $Z_{\hbar }(U,R)$ and inverses of elements in $Z_{\hbar }^{+}(U,R)$.

## Curvature

In this section we introduce a module of vector fields over $\widehat{\mathcal {C}}_\hbar$, together with a compatible connection. Given a metric *h*, it turns out that there exists a unique torsion-free and almost complex connection that is compatible with *h*. Moreover, the corresponding curvature tensor is computed as well as the Ricci and scalar curvature.

For the classical catenoid, parametrized by

$$\begin{aligned} \vec {x}(u,v) = \big (\cosh (u)\cos (v),\cosh (u)\sin (v),u\big ) \end{aligned}$$

the space of (complex) vector fields can be spanned by $\phi$ and $\bar{\phi }$, where

$$\begin{aligned} \phi = 2\partial \vec {x}= (\sinh (z),-i\cosh (z),1) \end{aligned}$$

with $z=u+iv$. Correspondingly, let $\{e_1,e_2,e_3\}$ denote the canonical basis of the free (right) module $(\widehat{\mathcal {C}}_\hbar )^3$, and set

$$\begin{aligned}&\Phi = e_1\Phi ^1+e_2\Phi ^2+e_3\Phi ^3\\&\bar{\Phi }= e_1(\Phi ^1)^*+e_2(\Phi ^2)^*+e_3(\Phi ^3)^*, \end{aligned}$$

where

$$\begin{aligned}&\Phi ^1 = \tfrac{1}{2}e^{\frac{1}{2}\hbar }(RW-R^{-1}W^{-1})\\&\Phi ^2 = -\tfrac{i}{2} e^{\frac{1}{2}\hbar }(RW+R^{-1}W^{-1})\\&\Phi ^3 = \mathbb {1}, \end{aligned}$$

and let $\mathcal {X}(\widehat{\mathcal {C}}_\hbar )$ denote the module generated by $\Phi$ and $\bar{\Phi }$. Note that

$$\begin{aligned}&\Phi ^1\sim \frac{1}{2}(e^ue^{iv}-e^{-u}e^{-iv})=\sinh (z)\\&\Phi ^2\sim -\frac{i}{2}(e^{u}e^{iv}+e^{-u}e^{-iv})=-i\cosh (z) \end{aligned}$$

when considering the formal correspondence $R\sim e^u$ and $W\sim e^{iv}$ as $\hbar \rightarrow 0$.

### Proposition 3.1

$\{\Phi ,\bar{\Phi }\}$ is a basis for $\mathcal {X}(\widehat{\mathcal {C}}_\hbar )$, which shows that $\mathcal {X}(\widehat{\mathcal {C}}_\hbar )$ is a free (right) $\widehat{\mathcal {C}}_\hbar$-module of rank 2.

### Proof

Let $a,b\in \widehat{\mathcal {C}}_\hbar$ and assume that $\Phi a+\bar{\Phi }b = 0$, which is equivalent to

$$\begin{aligned}&\Phi ^1a+(\Phi ^1)^*b = 0\\&\Phi ^2a+(\Phi ^2)^*b = 0\\&\Phi ^3a+(\Phi ^3)^*b = 0 \end{aligned}$$

By multiplying these equations from the left by $\Phi ^1,\Phi ^2,\Phi ^3$ respectively, and taking their sum, one obtains

$$\begin{aligned} \left( \Phi ^1(\Phi ^1)^*+\Phi ^2(\Phi ^2)^*+\Phi ^3(\Phi ^3)^*\right) b=0 \quad \Rightarrow \quad b=0 \end{aligned}$$

since

$$\begin{aligned}&\big (\Phi ^1\big )^2+\big (\Phi ^2\big )^2+\big (\Phi ^3\big )^2= \frac{1}{4}e^{\hbar }\Big ((RW-R^{-1}W^{-1})^2-(RW+R^{-1}W^{-1})^2\Big ) + \mathbb {1}\\&=\frac{1}{4}e^{\hbar }\big (RWRW-RWR^{-1}W^{-1}-R^{-1}W^{-1}RW+R^{-1}W^{-1}\big )\\&\quad -\frac{1}{4}e^{\hbar } \big (RWRW+R^{-1}W^{-1}R^{-1}W^{-1}+RWR^{-1}W^{-1}+R^{-1}W^{-1}RW\big )+\mathbb {1}\\&=-\,\frac{1}{2}e^{\hbar }\big (RWR^{-1}W^{-1}+R^{-1}W^{-1}RW\big )+\mathbb {1}=-\,\frac{1}{2}e^{\hbar }\big (e^{-\hbar }\mathbb {1}+e^{-\hbar }\mathbb {1}\big )+\mathbb {1}=0. \end{aligned}$$

Analogously, one may multiply the equations from the left with $(\Phi ^1)^*,(\Phi ^2)^*,(\Phi ^3)^*$ respectively, and find that their sum implies that $a=0$. Since, by definition, $\Phi$ and $\bar{\Phi }$ generate $\mathcal {X}(\widehat{\mathcal {C}}_\hbar )$, this shows that $\{\Phi ,\bar{\Phi }\}$ is indeed a basis for $\mathcal {X}(\widehat{\mathcal {C}}_\hbar )$. $\square$

Let *h* be a hermitian form (or metric) on $\mathcal {X}(\widehat{\mathcal {C}}_\hbar )$, i.e.

$$\begin{aligned}&h(X,Y+Z) = h(X,Y)+h(X,Z)\\&h(X,Y)^*= h(Y,X)\\&h(X,Ya) = h(X,Y)a \end{aligned}$$

for all $X,Y,Z\in \mathcal {X}(\widehat{\mathcal {C}}_\hbar )$ and $a\in \widehat{\mathcal {C}}_\hbar$. We will assume a diagonal metric on $\mathcal {X}(\widehat{\mathcal {C}}_\hbar )$ given by

$$\begin{aligned} h(\Phi ,\Phi ) = S,\qquad h(\bar{\Phi },\bar{\Phi })=T,\qquad h(\Phi ,\bar{\Phi }) = 0 \end{aligned}$$

with *S* and *T* being invertible, implying that the metric is non-degenerate. For instance, one may consider the induced metric from the free module by letting $h:(\widehat{\mathcal {C}}_\hbar )^3\times (\widehat{\mathcal {C}}_\hbar )^3\rightarrow \widehat{\mathcal {C}}_\hbar$ denote the bilinear form defined by

$$\begin{aligned} h(X,Y) = \sum _{i=1}^3(X^i)^*Y^i \end{aligned}$$

for $X=e_iX^i$ and $Y=e_iY^i$, for which one computes

$$\begin{aligned}&S = h(\Phi ,\Phi ) = \mathbb {1}+\tfrac{1}{2}e^{-\hbar }\big (R^2+R^{-2}\big )\\&T = h(\bar{\Phi },\bar{\Phi }) = \mathbb {1}+\tfrac{1}{2}e^{\hbar }\big (R^2+R^{-2}\big )\\&h(\Phi ,\bar{\Phi }) = h(\bar{\Phi },\Phi ) = 0. \end{aligned}$$

Note that $S,T\in Z_{\hbar }^{+}(U,R)$ (implying that they are invertible). We emphasize that in what follows, *S* and *T* are taken to be arbitrary invertible elements of $\widehat{\mathcal {C}}_\hbar$.

A connection on $\mathcal {X}(\widehat{\mathcal {C}}_\hbar )$ is a map $\nabla :\mathfrak {g}\times \mathcal {X}(\widehat{\mathcal {C}}_\hbar )\rightarrow \mathcal {X}(\widehat{\mathcal {C}}_\hbar )$ such that

$$\begin{aligned}&\nabla _d(\lambda X+Y) = \lambda \nabla _dX + \nabla _dY\\&\nabla _{\lambda d+d'}X = \lambda \nabla _dX + \nabla _{d'}X\\&\nabla _{d}(Xa) = \big (\nabla _dX\big )a+Xd(a), \end{aligned}$$

for $\lambda \in {\mathbb {C}}$, $X,Y\in \mathcal {X}(\widehat{\mathcal {C}}_\hbar )$, $d,d'\in \mathfrak {g}$, and $a\in \widehat{\mathcal {C}}_\hbar$. A connection is called *hermitian* if

$$\begin{aligned} dh(X,Y) = h(\nabla _{d^*}X,Y) + h(X,\nabla _dY), \end{aligned}$$

for $d\in \mathfrak {g}$ and $X,Y\in \mathcal {X}(\widehat{\mathcal {C}}_\hbar )$. Moreover, we say that $\nabla$ is *torsion-free* if

$$\begin{aligned} \nabla _{\partial }\bar{\Phi }=\nabla _{\bar{\partial }}\Phi . \end{aligned}$$

Let us introduce an almost complex structure $J:\mathcal {X}(\widehat{\mathcal {C}}_\hbar )\rightarrow \mathcal {X}(\widehat{\mathcal {C}}_\hbar )$ by setting

$$\begin{aligned}&J\Phi = i\Phi \\&J\bar{\Phi }= -i\bar{\Phi }\end{aligned}$$

and extending *J* to $\mathcal {X}(\widehat{\mathcal {C}}_\hbar )$ as a (right) $\widehat{\mathcal {C}}_\hbar$-module homomorphism. A connection is called *almost complex* if

$$\begin{aligned} (\nabla _d J)(X)\equiv \nabla _dJ(X)-J\nabla _d X = 0 \end{aligned}$$

for all $d\in \mathfrak {g}$ and $X\in \mathcal {X}(\widehat{\mathcal {C}}_\hbar )$.

With respect to the basis $\{\Phi ,\bar{\Phi }\}$, a connection on $\mathcal {X}(\widehat{\mathcal {C}}_\hbar )$ is given by choosing arbitrary $\Gamma ^{a}_{bc}\in \widehat{\mathcal {C}}_\hbar$ (for $a,b,c\in \{1,2\}$) and setting

$$\begin{aligned} \nabla _a X\equiv \nabla _{\partial _a}X = \Phi _b\partial _aX^b+\Phi _c\Gamma ^{c}_{ab}X^b \end{aligned}$$

where $\Phi _1=\Phi$, $\Phi _2=\bar{\Phi }$, $\partial _1=\partial$, $\partial _2=\bar{\partial }$ and $X=\Phi _aX^a$. Demanding that the connection is torsion-free immediately gives that $\Gamma ^a_{bc}=\Gamma ^a_{cb}$.

### Lemma 3.2

Let $\nabla$ be a connection on $\mathcal {X}(\widehat{\mathcal {C}}_\hbar )$ given as

$$\begin{aligned} \nabla _aX = \Phi _b\partial _aX^b+\Phi _c\Gamma ^{c}_{ab}X^b. \end{aligned}$$

The connection is almost complex if and only if

$$\begin{aligned}&\Gamma ^{2}_{11}=\Gamma ^{1}_{22}=0\\&\Gamma ^2_{21}=\Gamma ^{1}_{12}=0. \end{aligned}$$

### Proof

The condition for $\nabla$ to be almost complex is equivalent to

3.1 $$\begin{aligned}&\nabla _{\partial }(J\Phi ) - J\nabla _{\partial }\Phi = 0 \end{aligned}$$

3.2 $$\begin{aligned}&\nabla _{\bar{\partial }}(J\Phi ) - J\nabla _{\bar{\partial }}\Phi = 0 \end{aligned}$$

3.3 $$\begin{aligned}&\nabla _{\partial }(J\bar{\Phi }) - J\nabla _{\partial }\bar{\Phi }= 0 \end{aligned}$$

3.4 $$\begin{aligned}&\nabla _{\bar{\partial }}(J\bar{\Phi }) - J\nabla _{\bar{\partial }}\bar{\Phi }= 0. \end{aligned}$$

For an arbitrary connection $\nabla$, given by

$$\begin{aligned} \nabla _aX\equiv \nabla _{\partial _a}X = \Phi _b\partial _aX^b+\Phi _c\Gamma ^{c}_{ab}X^b, \end{aligned}$$

these equations are equivalent to 
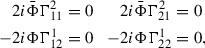
 which immediately gives the desired result since $\{\Phi ,\bar{\Phi }\}$ is a basis for $\mathcal {X}(\widehat{\mathcal {C}}_\hbar )$. $\square$

Thus, it follows from Lemma 3.2 that a connection is torsion-free and almost complex if and only if

3.5 $$\begin{aligned}&\nabla _\partial \Phi = \Phi \Gamma ^1,\quad \nabla _{\bar{\partial }}\bar{\Phi }= \bar{\Phi }\Gamma ^2,\quad \nabla _{\bar{\partial }}\Phi = \nabla _{\partial }\bar{\Phi }=0 \end{aligned}$$

for some $\Gamma ^1,\Gamma ^2\in \widehat{\mathcal {C}}_\hbar$. If, in addition, the connection is hermitian the connection coefficients will be uniquely fixed, as formulated in the next result.

### Theorem 3.3

There exists a unique hermitian torsion-free almost complex connection $\nabla$ on $\mathcal {X}(\widehat{\mathcal {C}}_\hbar )$, given by

$$\begin{aligned} \nabla _\partial \Phi&= \Phi S^{-1}\partial S\\ \nabla _{\bar{\partial }}\bar{\Phi }&= \bar{\Phi }T^{-1}\bar{\partial }T\\ \nabla _{\bar{\partial }}\Phi&= \nabla _\partial \bar{\Phi }= 0. \end{aligned}$$

### Proof

As noted in (3.5), an arbitrary torsion-free almost complex connection may be written as

$$\begin{aligned}&\nabla _\partial \Phi = \Phi \Gamma ^1,\quad \nabla _{\bar{\partial }}\bar{\Phi }= \bar{\Phi }\Gamma ^2,\quad \nabla _{\bar{\partial }}\Phi = \nabla _{\partial }\bar{\Phi }=0. \end{aligned}$$

To prove that the connection is compatible with the metric, one needs to show that

$$\begin{aligned}&\partial h(\Phi _a,\Phi _b) = h(\nabla _{\bar{\partial }}\Phi _a,\Phi _b)+h(\Phi _a,\nabla _{\partial }\Phi _b)\\&\bar{\partial }h(\Phi _a,\Phi _b) = h(\nabla _{\partial }\Phi _a,\Phi _b)+h(\Phi _a,\nabla _{\bar{\partial }}\Phi _b) \end{aligned}$$

for $a,b\in \{1,2\}$. For $a\ne b$, the above conditions are void since each term is separately zero. For $a=b=1$ one obtains

$$\begin{aligned} \partial S = S\Gamma ^1\quad \text {and}\quad \bar{\partial }S = (\Gamma ^1)^*S \end{aligned}$$

both giving $\Gamma ^1=S^{-1}\partial S$. Similarly, for $a=b=2$ one obtains $\Gamma ^2=T^{-1}\bar{\partial }T$. Thus, demanding that a connection is metric, torsion-free and almost complex uniquely fixes the connection components $\Gamma ^{a}_{bc}$. $\square$

The curvature $R(\partial _a,\partial _b)X=\nabla _a\nabla _bX-\nabla _b\nabla _aX$ is easily computed to be

3.6 $$\begin{aligned}&R(\partial ,\bar{\partial })\Phi = -\Phi \bar{\partial }\big (S^{-1}\partial S\big ) \end{aligned}$$

3.7 $$\begin{aligned}&R(\partial ,\bar{\partial })\bar{\Phi }= \bar{\Phi }\partial \big (T^{-1}\bar{\partial }T\big ) \end{aligned}$$

and since $\mathcal {X}(\widehat{\mathcal {C}}_\hbar )$ is a free module, one has uniquely defined curvature components $R(\partial _a,\partial _b)\Phi _c=\Phi _p{R^p}_{cab}$ given by

$$\begin{aligned}&{R^{1}}_{112}=-\bar{\partial }\big (S^{-1}\partial S\big )\qquad {R^2}_{212}=\partial \big (T^{-1}\bar{\partial }T\big )\\&{R^{1}}_{212}={R^{2}}_{112}=0. \end{aligned}$$

One may also proceed to define $R_{abpq}=h(\bar{\Phi }_a,R(\partial _p,\partial _q)\Phi _b)$, where $\bar{\Phi }_1=\bar{\Phi }$ and $\bar{\Phi }_2=\Phi$, giving

$$\begin{aligned}&R_{1212}=T\partial \big (T^{-1}\bar{\partial }T\big )\qquad R_{2112}=-S\bar{\partial }\big (S^{-1}\partial S\big )\\&R_{1112}=R_{2212}=0 \end{aligned}$$

and we note that $R_{abpq}$ does not enjoy the all the symmetries of the classical Riemann tensor due to the fact that $S\ne T$ in the noncommutative setting.

Furthermore, there are natural definitions of both Ricci and scalar curvature

$$\begin{aligned}&{\text {Ric}}_{ab} = {R^p}_{apb}\\&R = -T^{-1}R_{1212}S^{-1}+S^{-1}R_{2112}T^{-1} \end{aligned}$$

giving

$$\begin{aligned}&{\text {Ric}}_{12}=-\bar{\partial }\big (S^{-1}\partial S\big )\qquad {\text {Ric}}_{21}=-\partial \big (T^{-1}\bar{\partial }T\big )\\&{\text {Ric}}_{11}={\text {Ric}}_{22} = 0.\\&R = -\partial \big (T^{-1}\bar{\partial }T\big )S^{-1}-\bar{\partial }\big (S^{-1}\partial S\big )T^{-1} \end{aligned}$$

In all these expressions we note the appearance of “logarithmic” derivatives, in analogy with $\partial \bar{\partial }\ln (f)=\partial (f^{-1}\bar{\partial }f)$. For the unique connection in Theorem 3.3 one may define both gradient and divergence in a natural way.

### Definition 3.4

For $f\in \widehat{\mathcal {C}}_\hbar$ and $X=\Phi a+\bar{\Phi }b\in \mathcal {X}(\widehat{\mathcal {C}}_\hbar )$, define

$$\begin{aligned} \nabla f&= \Phi S^{-1}\bar{\partial }f+\bar{\Phi }T^{-1}\partial f\\ {\text {div}}(X)&= S^{-1}\partial (Sa)+T^{-1}\bar{\partial }(Tb)\\ \Delta (f)&= {\text {div}}(\nabla f). \end{aligned}$$

An element $f\in \widehat{\mathcal {C}}_\hbar$ is called *harmonic* if $\Delta (f)=0$.

### Proposition 3.5

If $$f\in \widehat{\mathcal {C}}_\hbar $$ then

$$\begin{aligned} \Delta (f) = (S^{-1}+T^{-1})\partial \bar{\partial }f. \end{aligned}$$

### Proof

The proof consists of a straight-forward computation:

$$\begin{aligned} {\text {div}}(\nabla f)&= {\text {div}}\big (\Phi S^{-1}\bar{\partial }f + \bar{\Phi }T^{-1}\partial f\big ) =S^{-1}\partial \big (SS^{-1}\bar{\partial }f\big )+T^{-1}\bar{\partial }\big (TT^{-1}\partial f\big )\\&= S^{-1}\partial \bar{\partial }f + T^{-1}\bar{\partial }\partial f = (S^{-1}+T^{-1})\partial \bar{\partial }f \end{aligned}$$

since $$[\partial ,\bar{\partial }]=0$$. $$\square $$

In classical geometry, the fact that the catenoid is a minimal surface may be characterized by demanding that the embedding coordinates $x^1,x^2,x^3$ are harmonic. A similar statement holds for $\widehat{\mathcal {C}}_\hbar$; namely, one notes that if

$$\begin{aligned}&X^1 = \tfrac{1}{2}e^{\tfrac{1}{2}\hbar }\big (RW+R^{-1}W^{-1}+(RW+R^{-1}W^{-1})^*\big )\\&X^2 = -\tfrac{i}{2}e^{\tfrac{1}{2}\hbar }\big (RW-R^{-1}W^{-1}-(RW-R^{-1}W^{-1})^*\big )\\&X^3 = U \end{aligned}$$

then $(X^i)^*= X^i$ and $\partial X^i=\Phi ^i$ for $i=1,2,3$, in analogy with the classical embedding coordinates into ${\mathbb {R}}^3$. One may readily check that the noncommutative embedding coordinates are harmonic

$$\begin{aligned} \Delta (X^1)&= (S^{-1}+T^{-1})\bar{\partial }\partial X^1 = (S^{-1}+T^{-1})\bar{\partial }\Phi ^1\\&= \tfrac{1}{2}e^{\tfrac{1}{2}\hbar }(S^{-1}+T^{-1})\bar{\partial }(RW-R^{-1}W^{-1})\\&= \tfrac{1}{2}e^{\tfrac{1}{2}\hbar }(S^{-1}+T^{-1}) \big (RW-RW+R^{-1}W^{-1}-R^{-1}W^{-1}\big ) = 0\\ \Delta (X^2)&= (S^{-1}+T^{-1})\bar{\partial }\partial X^2 = (S^{-1}+T^{-1})\bar{\partial }\Phi ^2\\&= \frac{i}{2}e^{\tfrac{1}{2}\hbar }(S^{-1}+T^{-1})\bar{\partial }(RW+R^{-1}W^{-1})\\&= \tfrac{1}{2}e^{\tfrac{1}{2}\hbar }(S^{-1}+T^{-1}) \big (RW-RW-R^{-1}W^{-1}+R^{-1}W^{-1}\big ) = 0\\ \Delta (X^3)&= (S^{-1}+T^{-1})\bar{\partial }\partial X^3 = (S^{-1}+T^{-1})\bar{\partial }(\tfrac{1}{2}\mathbb {1}) = 0. \end{aligned}$$

## Integration and total curvature

Let us introduce a concept of integration in analogy with integration on the classical catenoid. The total integral of a function on the catenoid, with respect to the induced metric can be computed in local coordinates as

$$\begin{aligned} \tau (f) =\int _{-\infty }^\infty \Bigg (\int _0^{2\pi }f(u,v)\cosh ^2(u)\text {d}v\Bigg )\text {d}u \end{aligned}$$

whenever the integral exists. For a function, expressible as

$$\begin{aligned} f(u,v) = \sum _{k\in {\mathbb {Z}}}f_k(u,e^u)e^{ikv} \end{aligned}$$

we note that $\tau (f)=\tau (f_0)$. To define a corresponding linear functional on $\widehat{\mathcal {C}}_\hbar$, we start by extending the map $\phi :Z_{\hbar }(U,R)\rightarrow C^\infty ({\mathbb {R}})$ (as defined in Sect. 2) to $\mathcal {F}^+_\hbar (U,R)$ by setting $\phi (p^{-1})=1/\phi (p)$ for $p\in Z_{\hbar }^{+}(U,R)$. Then, given $a\in \mathcal {C}_{\hbar }$

$$\begin{aligned} a = \sum _{k\in {\mathbb {Z}}}a_kW^k \end{aligned}$$

we set

$$\begin{aligned} \tau _0(a) = 2\pi \int _{-\infty }^\infty \phi (a_0)\text {d}u \end{aligned}$$

whenever the integral exists. Note that $\tau _0$ is in general not a trace.

Given a conformal metric of the type

$$\begin{aligned} h(\Phi ,\Phi ) = h(\bar{\Phi },\bar{\Phi }) = S\qquad h(\Phi ,\bar{\Phi }) = 0 \end{aligned}$$

where $S\in \mathcal {F}^+_\hbar (U,R)$ is invertible, one introduces an integral with respect to the corresponding volume form as

$$\begin{aligned} \tau _h(a) = 4\pi \int _{-\infty }^\infty \phi (a_0)\phi (S)\,\text {d}u. \end{aligned}$$

The extra factor of two is introduced for convenience due to the fact that for a conformal metric with $h(\partial ,\partial )=h(\bar{\partial },\bar{\partial })=s$ one finds that $g(\partial _u,\partial _u)=g(\partial _v,\partial _v)=2s$. As an illustration of the above concepts, let us compute the noncommutative total curvature, i.e. the integral of the Gaussian curvature (defined to be half of the scalar curvature). From Sect. 3 one finds the Gaussian curvature

$$\begin{aligned} K = -\frac{1}{4}\partial _u\big (S^{-1}\partial _u S\big )S^{-1} \end{aligned}$$

since $\partial S = \bar{\partial }S=\tfrac{1}{2}\partial _u S$ when $S\in \mathcal {F}^+_\hbar (U,R)$, and the corresponding integral gives

$$\begin{aligned} \tau _h(K) = -\pi \int _{-\infty }^\infty \partial _u\big (s^{-1}\partial _us\big )\text {d}u =-\pi \Big [s^{-1}\partial _u s\Big ]_{-\infty }^\infty , \end{aligned}$$

where $s=\phi (S)$. For instance, choosing a metric in analogy with the induced metric from ${\mathbb {R}}^3$

$$\begin{aligned}&S = \frac{1}{4}(R+R^{-1})^2\quad \Rightarrow \quad s=\phi (S)=\cosh ^2(u)\quad \Rightarrow \quad \\&\tau _h(K) = -\pi \Big [2\tanh (u)\Big ]_{-\infty }^{\infty }=-4\pi , \end{aligned}$$

in accordance with the classical result.

## Bimodules

In this section we will introduce classes of left and right modules over $\mathcal {C}_{\hbar }$, as well as study compatibility conditions for bimodules. Moreover, constant curvature connections are introduced and their relations to the bimodule structure is discussed.

Let $C^\infty _0({\mathbb {R}}\times {\mathbb {Z}})$ denote the space of complex valued smooth functions on ${\mathbb {R}}\times {\mathbb {Z}}$ with compact support (in both variables), together with the inner product

5.1 $$\begin{aligned} \left\langle \xi ,\eta \right\rangle = \sum _{k=-\infty }^\infty \int _{-\infty }^\infty \xi (x,k)\bar{\eta }(x,k)\text {d}x. \end{aligned}$$

### Proposition 5.1

Let $\lambda _0,\lambda _1,\varepsilon \in {\mathbb {R}}$ and $r\in {\mathbb {Z}}$. If $\lambda _0\varepsilon +\lambda _1r=-\hbar$ then

$$\begin{aligned} (W\xi )(x,k)&=\xi (x-\varepsilon ,k-1)\\ (W^{-1}\xi )(x,k)&=\xi (x+\varepsilon ,k+1)\\ (R\xi )(x,k)&= e^{\lambda _0x+\lambda _1k}\xi (x,k)\\ (R^{-1}\xi )(x,k)&= e^{-\lambda _0x-\lambda _1k}\xi (x,k)\\ (U\xi )(x,k)&=(\lambda _0x+\lambda _1k)\xi (x,k) \end{aligned}$$

for $\xi \in C^\infty _0({\mathbb {R}}\times {\mathbb {Z}})$, defines a left $\mathcal {C}_{\hbar }$-module structure on $C^\infty _0({\mathbb {R}}\times {\mathbb {Z}})$. Correspondingly,

$$\begin{aligned} (\xi W)(x,k)&= \xi (x-\varepsilon ',k-r')\\ (\xi W^{-1})(x,k)&= \xi (x+\varepsilon ',k+r')\\ (\xi R)(x,k)&= e^{\mu _0x+\mu _1k}\xi (x,k)\\ (\xi R^{-1})(x,k)&= e^{-\mu _0x-\mu _1 k}\xi (x,k)\\ (\xi U)(x,k)&= (\mu _0x+\mu _1k)\xi (x,k) \end{aligned}$$

defines a right $\mathcal {C}_{\hbar }$-module structure on $C^\infty _0({\mathbb {R}}\times {\mathbb {Z}})$ if $\mu _0,\mu _1,\varepsilon '\in {\mathbb {R}}$ and $r'\in {\mathbb {Z}}$ such that $\mu _0\varepsilon '+\mu _1r'=\hbar$. Moreover, both the left and right module structures are compatible with the inner product; i.e.

$$\begin{aligned} \left\langle a\xi ,\eta \right\rangle =\left\langle \xi ,a^*\eta \right\rangle \quad \text {and}\quad \left\langle \xi a,\eta \right\rangle = \left\langle \xi ,\eta a^* \right\rangle \end{aligned}$$

for $$a\in \mathcal {C}_{\hbar }$$ and $$\xi ,\eta \in C^\infty _0({\mathbb {R}}\times {\mathbb {Z}})$$.

### Proof

Let us show that the above definitions define a left module structure. The right module structure is checked in an analogous way.

It follows immediately from the definitions that

$$\begin{aligned} WW^{-1}\xi&=\xi ,\quad W^{-1}W\xi = \xi ,\quad RR^{-1}\xi =\xi ,\quad R^{-1}R\xi = \xi \\ {[}R,U]\xi&=0,\quad [R^{-1},U]\xi = 0. \end{aligned}$$

Thus, it remains to check the following relations:

$$\begin{aligned}&WR=e^{\hbar }RW\\&WU=UW+\hbar W. \end{aligned}$$

One gets

$$\begin{aligned}&WR\xi (x,k) -e^\hbar RW\xi (x,k)= We^{\lambda _0x+\lambda _1k}\xi (x,k) -e^\hbar R\xi (x-\varepsilon ,k-r)\\&\quad =e^{\lambda _0(x-\varepsilon )+\lambda _1(k-r)}\xi (x-\varepsilon ,k-r) -e^{\hbar }e^{\lambda _0x+\lambda _1k}\xi (x-\varepsilon ,k-r)\\&\quad =e^{\lambda _0x+\lambda _1k} \big (e^{-\lambda _0\varepsilon -\lambda _1r}-e^{\hbar }\big )\xi (x-\varepsilon ,k-r)=0, \end{aligned}$$

by using that $\lambda _0\varepsilon +\lambda _1r=-\hbar$. Finally, one computes

$$\begin{aligned} WU&\xi (x,k)-UW\xi (x,k)-\hbar W\xi (x,k)\\&=W(\lambda _0x+\lambda _1k)\xi (x,k) -U\xi (x-\varepsilon ,k-r)-\hbar \xi (x-\varepsilon ,k-r)\\&=\big (\lambda _0(x-\varepsilon )+\lambda _1(k-r)\big )\xi (x-\varepsilon ,k-r) -(\lambda _0x+\lambda _1k)\xi (x-\varepsilon ,k-r)\\&\qquad -\,\hbar \xi (x-\varepsilon ,k-r)\\&=\big (-\lambda _0\varepsilon -\lambda _1r-\hbar \big )\xi (x-\varepsilon ,k-r)=0, \end{aligned}$$

again using that $\lambda _0\varepsilon +\lambda _1r=-\hbar$. It is now straightforward to check that the module structure is compatible with the inner product; e.g.

$$\begin{aligned} \left\langle W\xi ,\eta \right\rangle = \sum _{k=-\infty }^\infty \int _{-\infty }^\infty \xi (x-\varepsilon ,k-r)\bar{\eta }(x,k)\text {d}x \end{aligned}$$

which, by setting $l=k-r$ and $y=x-\varepsilon$, becomes

$$\begin{aligned} \left\langle W\xi ,\eta \right\rangle = \sum _{l=-\infty }^\infty \int _{-\infty }^\infty \xi (y,l)\bar{\eta }(y+\varepsilon ,l+r)\text {d}y=\left\langle \xi ,W^{-1}\eta \right\rangle =\left\langle \xi ,W^*\eta \right\rangle , \end{aligned}$$

and similar computations are carried out for the remaining generators. $\square$

In order for $C^\infty _0({\mathbb {R}}\times {\mathbb {Z}})$ to be a $\mathcal {C}_{\hbar }-\mathcal {C}_{\hbar '}$-bimodule, one has to demand that the two structures in Proposition 5.1 are compatible; i.e. that $A(\xi B)=(A\xi )B$ for all $A\in \mathcal {C}_{\hbar }$ and $B\in \mathcal {C}_{\hbar '}$. This induces certain compatibility conditions on the parameters, as formulated in the next result.

### Proposition 5.2

Let $\lambda _0,\lambda _1,\varepsilon ,\varepsilon '\in {\mathbb {R}}$ and $r,r'\in {\mathbb {Z}}$ such that $\lambda _0\varepsilon +\lambda _1r=-\hbar$ and $\mu _0\varepsilon '+\mu _1r'=-\hbar '$. If $\lambda _0\varepsilon '+\lambda _1r'=0$ and $\mu _0\varepsilon +\mu _1r=0$ then $C^\infty _0({\mathbb {R}}\times {\mathbb {Z}})$ is a $\mathcal {C}_{\hbar }-\mathcal {C}_{\hbar '}$-bimodule.

### Proof

To prove that $C^\infty _0({\mathbb {R}}\times {\mathbb {Z}})$ is a bimodule, one has to show that the left and right module structures given in Proposition 5.1 are compatible; i.e.

$$\begin{aligned} \big ((A\xi )B\big )(x,k) = \big (A(\xi B)\big )(x,k) \end{aligned}$$

for all $A\in \mathcal {C}_{\hbar }$ and $B\in \mathcal {C}_{\hbar '}$. It is enough to check compatibility for the generators, for instance

$$\begin{aligned} \big ((W\xi )R\big )(x,k)&-\big (W(\xi R)\big )(x,k)= e^{\mu _0x+\mu _1k}(W\xi )(x,k)-(\xi R)(x-\varepsilon ,k-r)\\&=\big (e^{\mu _0x+\mu _1k}-e^{\mu _0(x-\varepsilon )+\mu _1(k-r)}\big )\xi (x-\varepsilon ,k-r) =0 \end{aligned}$$

since $\mu _0\varepsilon +\mu _1r = 0$. Similarly, one gets

$$\begin{aligned} \big ((R\xi )W\big )(x,k)&-\big (R(\xi W)\big )(x,k)= (R\xi )(x-\varepsilon ',k-r')-e^{\lambda _0x+\lambda _1k}(\xi W)(x,k)\\&=\big (e^{\lambda _0(x-\varepsilon ')+\lambda _1(k-r')}-e^{\lambda _0x+\lambda _1k}\big )\xi (x-\varepsilon ',k-r')=0 \end{aligned}$$

since $\lambda _0\varepsilon '+\lambda _1r'=0$. The remaining compatibility conditions may be checked in an analogous way. $\square$

Note that it possible to obtain a $\mathcal {C}_{\hbar }-\mathcal {C}_{\hbar '}$-bimodule for arbitrary $\hbar ,\hbar '\in {\mathbb {R}}$; namely, for $\varepsilon \ne \varepsilon '$ one may set

$$\begin{aligned} r&= r' = 1\qquad \lambda _0=-\frac{\hbar }{\varepsilon -\varepsilon '}\qquad \mu _0=-\frac{\hbar '}{\varepsilon -\varepsilon '}\\ \lambda _1&= \frac{\hbar \varepsilon '}{\varepsilon -\varepsilon '}\qquad \mu _1 = \frac{\hbar '\varepsilon }{\varepsilon -\varepsilon '}, \end{aligned}$$

fulfilling the requirements of Proposition 5.2.

Define linear maps $\nabla _u,\nabla _v:C^\infty _0({\mathbb {R}}\times {\mathbb {Z}})\rightarrow C^\infty _0({\mathbb {R}}\times {\mathbb {Z}})$ via

5.2 $$\begin{aligned} (\nabla _u\xi )(x,k) = \alpha \frac{d\xi }{\text {d}x}(x,k) \qquad \text {and}\qquad (\nabla _v\xi )(x,k) = \beta x\xi (x,k) \end{aligned}$$

for $\alpha ,\beta \in {\mathbb {C}}$. It is straightforward to check that

$$\begin{aligned}&\nabla _u(a\xi ) = a\nabla _u\xi + (\partial _u a)\xi \\&\nabla _v(a\xi ) = a\nabla _v\xi + (\partial _v a)\xi \end{aligned}$$

for all $a\in \mathcal {C}_{\hbar }$ if and only if $\alpha =1/\lambda _0$ and $\beta =i/\varepsilon$. Similarly, it holds that

$$\begin{aligned}&\nabla _u(\xi a) = (\nabla _u\xi )a + \xi (\partial _u a)\\&\nabla _v(\xi a) = (\nabla _v\xi )a + \xi (\partial _v a) \end{aligned}$$

for all $a\in \mathcal {C}_{\hbar '}$ if and only if $\alpha =1/\mu _0$ and $\beta =i/\varepsilon '$. Thus, (5.2) defines a left resp. right connection on $C^\infty _0({\mathbb {R}}\times {\mathbb {Z}})$ of constant curvature $\alpha \beta$; i.e.

$$\begin{aligned} \nabla _u\nabla _v\xi -\nabla _v\nabla _u\xi = \alpha \beta \xi . \end{aligned}$$

By choosing suitable parameters, one obtains a bimodule connection.

### Proposition 5.3

Assume that $C^\infty _0({\mathbb {R}}\times {\mathbb {Z}})$ is a $\mathcal {C}_{\hbar }-\mathcal {C}_{\hbar '}$-bimodule as in Proposition 5.2, and that

$$\begin{aligned}&(\nabla _u\xi )(x,k) = \frac{1}{\lambda _0}\frac{d\xi }{\text {d}x}(x,k)\\&(\nabla _v\xi (x,k)) = \frac{i}{\varepsilon }x\xi (x,k) \end{aligned}$$

is a bimodule connection on $C^\infty _0({\mathbb {R}}\times {\mathbb {Z}})$.

1. If $$\hbar =\hbar '$$ then $$\hbar =\hbar '=0$$,
2. if $\hbar \ne \hbar '$ then $\hbar /\hbar '\in {\mathbb {Q}}$ and
  $$\begin{aligned}&\lambda _0=\mu _0=\frac{\hbar r'}{\varepsilon (r-r')}\qquad \lambda _1=-\frac{\hbar }{r-r'}\qquad \mu _1 = -\frac{\hbar '}{r-r'} \end{aligned}$$
  for arbitrary $\varepsilon =\varepsilon '\in {\mathbb {R}}$ and $r,r'\in {\mathbb {Z}}$ such that $r/r'=\hbar /\hbar '$. Moreover,
  $$\begin{aligned} \nabla _u\nabla _v\xi (x,k)-\nabla _v\nabla _u\xi (x,k) = i\frac{\hbar -\hbar '}{\hbar \hbar '}\xi (x,k). \end{aligned}$$

### Proof

For a bimodule connection one must necessarily have $\lambda _0=\mu _0$ and $\varepsilon =\varepsilon '$. Together with the conditions in Proposition 5.2 one obtains the equations

5.3 $$\begin{aligned}&\lambda _0\varepsilon +\lambda _1r=-\hbar \end{aligned}$$

5.4 $$\begin{aligned}&\lambda _0\varepsilon +\mu _1r' = \hbar ' \end{aligned}$$

5.5 $$\begin{aligned}&\lambda _0\varepsilon +\lambda _1r'=0 \end{aligned}$$

5.6 $$\begin{aligned}&\lambda _0\varepsilon +\mu _1r = 0. \end{aligned}$$

Since $\lambda _0,\varepsilon \ne 0$ (in order for the bimodule connection to be defined), Eqs. (5.5) and (5.6) imply that $r,r'\ne 0$. Thus, one may solve these equations as

5.7 $$\begin{aligned} \lambda _1 = -\frac{\lambda _0\varepsilon }{r'}\qquad \mu _1 = -\frac{\lambda _0\varepsilon }{r}. \end{aligned}$$

Inserting (5.7) into (5.3) and (5.4) gives

5.8 $$\begin{aligned}&\lambda _0\varepsilon \Big (1-\frac{r}{r'}\Big ) =-\hbar \end{aligned}$$

5.9 $$\begin{aligned}&\lambda _0\varepsilon \Big (1-\frac{r'}{r}\Big ) = \hbar ' \end{aligned}$$

Now, assume that $\hbar =\hbar '$. Summing (5.8) and (5.9) yields

$$\begin{aligned} \frac{r}{r'}+\frac{r'}{r} = 2, \end{aligned}$$

which has the unique solution $r=r'$, implying that $\hbar =\hbar '=0$ via (5.8). This proves the first part of the statement. Next, assume that $\hbar \ne \hbar '$.

First we note that neither $\hbar$ nor $\hbar '$ can be zero, since that implies [by (5.8) and (5.9)] that $\hbar =\hbar '=0$ contradicting the assumption that $\hbar \ne \hbar '$. Thus, we can assume that $\hbar ,\hbar '\ne 0$, which implies that $r\ne r'$ [again, by (5.8) and (5.9)]. Solving (5.8) for $\lambda _0$ gives

5.10 $$\begin{aligned} \lambda _0 = \frac{\hbar r'}{\varepsilon (r-r')} \end{aligned}$$

which, when inserted in (5.9), gives

$$\begin{aligned} \frac{r}{r'} = \frac{\hbar }{\hbar '}. \end{aligned}$$

Hence, the quotient $\hbar /\hbar '$ is necessarily rational, since $r,r'\in {\mathbb {Z}}$, and inserting (5.10) into (5.7) yields

$$\begin{aligned} \lambda _1 = -\frac{\hbar }{r-r'}\qquad \mu _1 = -\frac{\hbar '}{r-r'}. \end{aligned}$$

Finally,

$$\begin{aligned} \lambda _0\varepsilon = \frac{\hbar r'}{r-r'} =\frac{\hbar \frac{r\hbar '}{\hbar }}{r-\frac{r\hbar '}{\hbar }} =\frac{\hbar \hbar '}{\hbar -\hbar '} \end{aligned}$$

giving

$$\begin{aligned} \nabla _u\nabla _v\xi -\nabla _v\nabla _u\xi =\frac{i}{\lambda _0\varepsilon }\xi =i\frac{\hbar -\hbar '}{\hbar \hbar '}\xi . \end{aligned}$$

$$\square $$

It is noteworthy that the curvature of the bimodule connection only depends on $\hbar$ and $\hbar '$ and is consequently independent of the particular choice of parameters that defines the bimodule. For the noncommutative torus, one proceeds to define compatible left and right hermitian structures, which implies first of all that the modules are projective and secondly, that certain torus algebras for different values of $\theta$ are Morita equivalent. It would be interesting to obtain similar results for the noncommutative catenoid.
